# Invasive micropapillary carcinoma of the breast had poor clinical characteristics but showed no difference in prognosis compared with invasive ductal carcinoma

**DOI:** 10.1186/s12957-016-0960-z

**Published:** 2016-08-05

**Authors:** Guanqiao Li, Shiping Yang, Jia Yao, Zhenping Wang, Guangyu Yao, Mingfeng Liu, Changsheng Ye

**Affiliations:** 1 Breast Center, Nanfang Hospital, Southern Medical University, Guangzhou, Guangdong 510515 People’s Republic of China; 2 Department of Radiation Oncology, Hainan Province People’s Hospital, Haikou, Hainan People’s Republic of China; 3 Department of Breast Surgery, Hainan Province People’s Hospital, Haikou, Hainan 570311 People’s Republic of China; 4 Department of Radiology, Hainan Province People’s Hospital, Haikou, Hainan 570311 People’s Republic of China

**Keywords:** Breast, Invasive micropapillary carcinoma, Invasive ductal carcinoma, Clinical characteristics, Survival, Retrospective study

## Abstract

**Background:**

It is controversial for prognosis of invasive micropapillary carcinoma (IMPC) compared with invasive ductal carcinoma (IDC) of the breast. To better understand the difference between IMPC and IDC prognoses, we conducted this retrospective study.

**Methods:**

Data from 33 patients with IMPC were retrospectively reviewed, and the clinicopathologic characteristics and survival status were compared with those of 347 patients with IDC who were treated during the same period.

**Results:**

The IMPC cases were of larger tumor size, greater proportion of nodal involvement, and an increased incidence of lymphovascular invasion compared with IDC cases. The overall survival (OS), local relapse-free survival (LRFS), distant metastasis-free survival (DMFS), and failure-free survival (FFS) rates were not significantly different between IMPC and IDC. The 3-year OS rate was 97 vs 94.2 % for the IMPC and IDC patients, respectively. The 3-year FFS rate was 87.9 vs 86.2 % for the IMPC and IDC patients, respectively. For IMPC patients, the 3-year LRFS rate was 93.9 % and in IDC patients was 89.0 %. The 3-year DMFS rates of IMPC patients was 90.9 % and IDC patients was 89 %.

**Conclusions:**

IMPC had poor clinical characteristics, but it showed no difference in OS, FFS, LRFS, and DMFS compare with IDC.

## Background

Breast cancer now represents the most common female malignancy in both the developing and developed world, and is the primary cause of death among women globally [1]. Invasive micropapillary carcinoma (IMPC) was first described by Siriaunkgul and Tavassoli as a rare variant of invasive breast carcinoma characterized by pseudopapillary and tubuloalveolar arrangement of tumor cell clusters in sponge-like, clear empty spaces, mimicking extensive lymphatic invasion [2].

As described by Luna More et al., IMPC is characterized by small, tightly cohesive groups of neoplastic cells within well-delineated clear spaces resembling lymphatic vessels [3].

The incidence of IMPC ranges from 3 to 6 % of all primary breast cancers [4]. Due to the low incidence of this breast cancer variant, most studies examining IMPC have small sample sizes; the clinicopathological characteristics and the clinical prognostic factors of invasive micropapillary carcinoma are therefore not well understood.

It is an important subtype due to its unique features such as high proclivity to lymphovascular invasion, lymph node metastasis, local recurrence, and distant metastasis, thus exhibiting a more aggressive behavior with a poorer prognosis than invasive ductal carcinoma (IDC) [4–8]. However, recently, it has been reported that this carcinoma IMPC has a similar or favorable prognosis compared with IDC [9, 10].

There is controversy about the prognosis of IMPC of the breast. Therefore, greater understanding about these rare tumors is urgent. The aims of this study were to investigate the clinicopathologic characteristics, treatment patterns, and the clinical outcomes compared with IDC in Hainan Island of South China. Moreover, the objective of this article is to draw attention to summarizing the survival rate of IMPC compared with IDC among the similar literatures.

## Methods

We conducted a retrospective study of breast cancer patients who were treated at the Hainan Province People’s Hospital between January 2010 and December 2012. This study was approved by the institutional review board and ethics committee of our hospital. In these periods, a total of 525 patients had operations for breast cancers in this institute. Of them, 33 patients (6.3 %) were diagnosed with IMPC (including pure and mixed type) and 347 patients were diagnosed with pure IDC. All IMPC cases included in the study displayed a micropapillary tumor component that was in accordance with the morphological criteria described in the WHO histological classification of tumors of the breast [11]. These patients were compared with 347 patients with pure IDC who were treated during the same period. Of the 33 IMPC cases, 16 patients (48.5 %) were identified as having pure IMPC, whereas 17 patients (51.5 %) had mixed IMPC. We reviewed clinicopathologic factors, immunohistochemistries of biologic factors such as estrogen receptor (ER), progesterone receptor (PR), human epidermal growth factor receptor 2 (HER2), and treatment modalities (type of operation, use of chemotherapy, radiation therapy, and hormone therapy). The pathologic tumor stage was assessed according to the sixth American Joint Committee on Cancer (AJCC) staging system [12]. All patients were followed up by our department at 3-month intervals for the first 2 years, every 6 months for 3–5 years, and annually thereafter. All events were measured from the date of commencement of operation. The following end points (time to the first defining event) were assessed: overall survival (OS), failure-free survival (FFS), local relapse-free survival (LRFS), and distant metastasis-free survival (DMFS).

### Statistical analysis

The clinicopathological parameters of the different subgroups were compared using Pearson’s chi-square test; Fisher’s exact test was used when needed. Survival curves were determined and plotted using the Kaplan-Meier method and group differences in survival time were investigated by log-rank test. *P* values less than 0.05 was considered statistically significant. Hazard ratios (HR) were presented with 95 % confidence intervals. SPSS for Windows (version 16.0, SPSS Inc., Chicago, IL, USA) was used for all statistical analyses.

## Results

### The clinicopathological characteristics of patients with IMPC and IDC

A total of 33 patients with IMPC of the breast were identified in our database. At the same time, 347 patients with IDC were also identified. The clinico-pathologic characteristics of all patients are summarized in Table 1.

**Table 1:** Baseline characteristics and treatment patterns for IMPC and IDC

	IMPC (*n* = 33)	IDC (*n* = 347)	P*
Age (year)			0.885
≤45	11 (33.3 %)	120 (34.6 %)	
>45	22 (66.7 %)	227 (65.4 %)	
Family history			0.179
Yes	2 (6.1 %)	7 (2.0 %)	
No	17 (21.5 %)	340 (98.0 %)	
ER status			0.011
Positive	27 (81.8 %)	206 (59.4 %)	
Negative	6 (18.2 %)	141 (40.6 %)	
PR status			0.123
Positive	25 (75.8 %)	209 (62.6 %)	
Negative	8 (24.2 %)	125 (37.4 %)	
Her2 status			0.479
Positive	6 (18.8 %)	82 (24.3 %)	
Negative	26 (81.2 %)	255 (75.7 %)	
Unknown	1	10	
Subtype			0.006
Luminal	29 (87.9 %)	223 (64.3 %)	
Non-luminal	4 (12.1 %)	124 (35.7 %)	
T classification			0.044
T1–T2	25 (75.8 %)	309 (89.0 %)	
T3–T4	8 (24.2 %)	38 (11.0 %)	
N classification			<0.001
N0	7 (21.2 %)	186 (53.8 %)	
N1–N3	26 (78.2 %)	160 (46.2 %)	
Unknown	0	1	
Staging			<0.001
I–II	15 (45.5 %)	262 (76.2 %)	
III	18 (54.5 %)	82 (23.8 %)	
Unknown	0	3	
Operation methods			0.392
BCS	0 (0 %)	19 (5.5 %)	
Mastectomy	33 (100 %)	328 (94.5 %)	
Adjuvant chemotherapy			0.709
Yes	32 (97 %)	321 (93.3 %)	
No	1 (3.0 %)	23 (6.7 %)	
Unknown	0	3	
Radiotherapy			0.146
Yes	14 (42.4 %)	104 (30.1 %)	
No	19 (57.6 %)	241 (69.9 %)	
Unknown	0	2	
Hormone therapy			0.026
Yes	28 (84.8 %)	227 (65.8 %)	
No	5 (15.2 %)	118 (34.2 %)	
Unknown	0	2	
Neoadjuvant chemotherapy			0.128
Yes	4 (12.1 %)	19 (5.5 %)	
No	29 (87.9 %)	328 (94.5 %)	
Trastuzumab			0.754
Yes	2 (6.1 %)	33 (9.5 %)	
No	31 (93.9 %)	314 (90.5 %)	
Lymphovascular invasion			<0.001
Yes	6 (18.2 %)	2 (0.6 %)	
No	27 (81.8 %)	345 (99.4 %)	
Nerve invasion			0.007
Yes	2 (6.1 %)	0 (0 %)	
No	3193.9 %)	347 (100 %)	

*IMPC* invasive micropapillary carcinoma, *IDC* invasive ductal carcinoma, *ER* estrogen receptor, *PR* progesterone receptor

* All P values calculated by two-sided x2 test

When comparing staging at presentation, IMPC patients had more T3 or T4 tumors (*P* = 0.044), a higher percentage of N1-3 nodal involvement (*P* < 0.001). IMPC patients had a higher incidence of lymphovascular invasion (*P* < 0.001) and nerve invasion (*P* = 0.007) compared with IDC patients.

Furthermore, IMPC patients had a larger proportion with luminal subtype than IDC patients (*P* = 0.006). Expressions of ER were significantly higher (*P* = 0.011), and expressions of PR were slight higher (*P* = 0.123) in IMPC than in IDC. Expressions of Her2 were not statistically significantly different in the IMPC and IDC cases. Hormone therapy was significantly higher in IMPC patients. Chemotherapy and radiotherapy were slightly higher in the IMPC group but not statistically significant. In addition, there were no significant differences in age, family history, and Her2 status.

### Survival of patients with IMPC and IDC

**Fig. 1:**
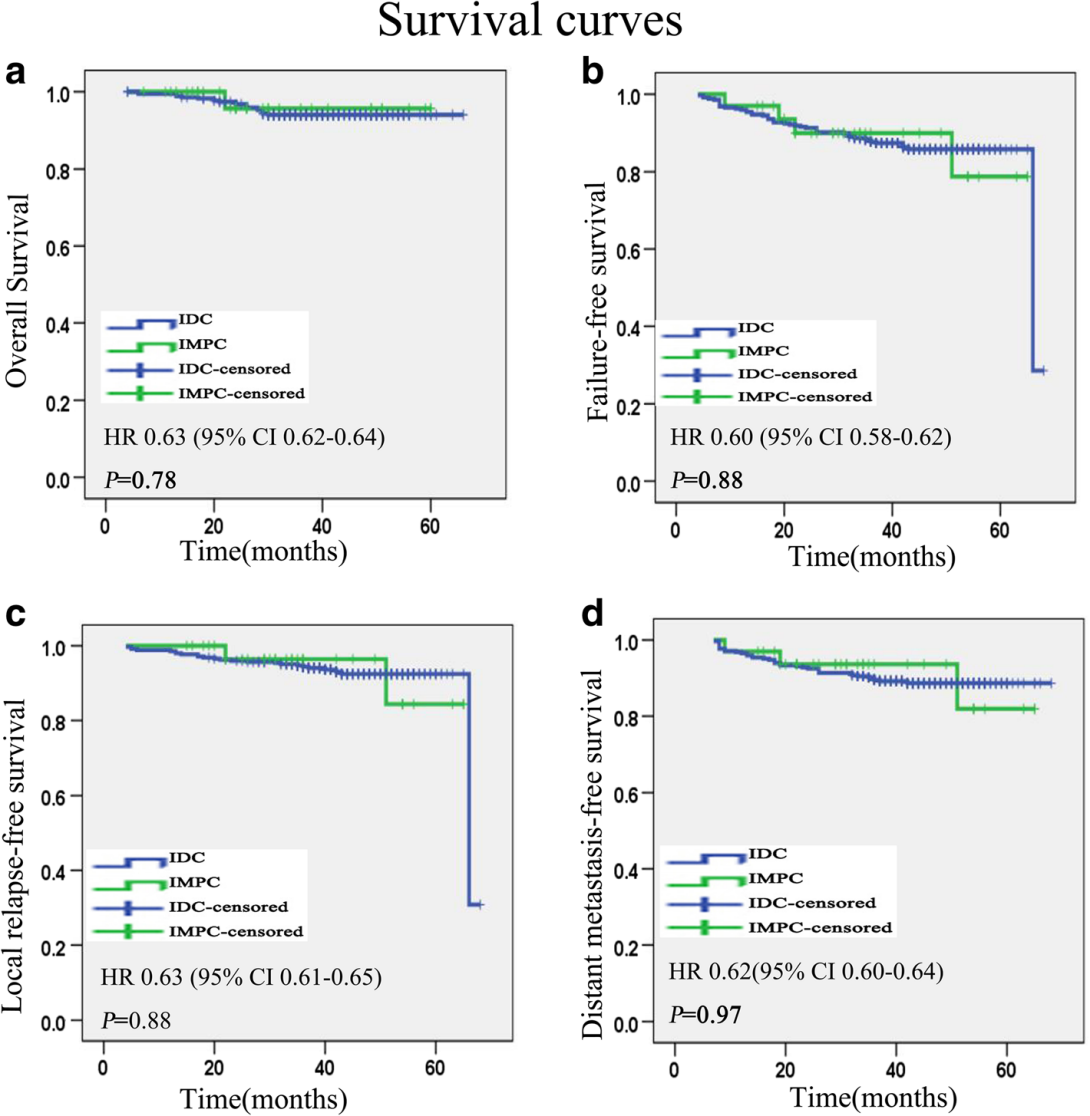
Kaplan-Meier survival curves for 33 patients with IMPC and 347 patients with IDC. Overall survival (**a**), failure-free survival (**b**), local relapse-free survival (**c**), and distant metastasis-free survival (**d**). *P* values were calculated with the unadjusted log-rank test. *IMPC* invasive micropapillary carcinoma, *IDC* invasive ductal carcinoma

The median follow-up duration was 39 months for all patients (range 6 to 66). The OS, FFS, LRFS, and DMFS rates were not significantly different between IMPC and IDC. The 3-year OS rate was 97 vs 94.2 % for the IMPC and IDC patients, respectively (*P* = 0.78) (Fig. 1a). The 3-year FFS rate was 87.9 vs 86.2 % for the IMPC and IDC patients, respectively (*P* = 0.88) (Fig. 1b). For IMPC patients, the 3-year LRFS rate was 93.9 %, and in IDC patients, it was 89.0 % (*P* = 0.88) (Fig. 1c). The 3-year DMFS rates of IMPC patients was 90.9 % and IDC patients was 89.0 % (*P* = 0.97) (Fig. 1d).

### Failure pattern

The 55 patients with treatment failure are listed in Table 2. The IMPC group had four patients that developed treatment failure: there was relapsing event in one patient, distant metastatic event in two patients (both of them had developed multi-organ metastasis), and both distant metastasis and recurrence in one patient.

**Table 2:** Patterns of failure in the IMPC and IDC patients after treatment

Patterns of failure	IMPC (*n* = 33)	IDC (*n* = 347)
Recurrence	*n* (%)	*n* (%)
Primary recurrence	1 (3 %)	9(2.6 %)
Nodal recurrence	0 (0 %)	2 (0.6 %)
Distant metastasis	*n* (%)	*n* (%)
Bone metastasis	0 (0 %)	10 (2.9 %)
Lung metastasis	0 (0 %)	5 (1.4 %)
Liver metastasis	0 (0 %)	2 (0.6 %)
Mediastina metastasis	0 (0 %)	0 (0.0 %)
Multiple metastasis	2 (6.1 %)	9 (2.6 %)
Distant metastasis, primary and/or nodal recurrence	1 (3 %)	14 (4 %)

*IMPC* invasive micropapillary carcinoma, *IDC* invasive ductal carcinoma

In IDC group, 51 patients in all had developed treatment failure: there was relapsing event in 11 patients, distant metastatic event in 26 patients and 14 patients had both distant metastasis and recurrence. Seventeen patients had developed distant metastasis in a single organ: ten cases in bone, five cases in lung, and two cases in liver. Nine patients had developed multi-organ metastasis.

## Discussion

IMPC is a rare pathological subtype of breast cancer, and the pure variant of IMPC is even rarer. Previous studies have shown that most patients (80~86 %) had mixed IMPC [7, 13]. In our study, most patients (57.6 %) had pure IMPC, whereas only 42.4 % had the mixed form.

Previous studies have shown that IMPC usually presents with a higher TNM stage and is associated with lymphovascular invasion and a higher propensity for lymph node metastases [4–8]. In our study, IMPC patients had more T3 or T4 tumors, a higher percentage of N1–N3 nodal involvement, and a higher incidence of lymphovascular invasion and nerve invasion compared with IDC patients, which is consistent with previous reports.

Most studies reported higher rate of ER positivity than the IDC comparison group (Table 3), and only two studies showed lower or similar rate of ER positivity (Table 3). In our study, the high percentages of ER and PR positivity in IMPCs (81.8 and 75.8 %, respectively) are in accordance with other reports [13–15]. Recently, Rodrigues’ study demonstrated the nuclear localization of epidermal growth factor receptor (EGFR) in the canine spontaneous model of IMPC of the mammary gland. This finding could be useful for EGFR as a predictive biomarker of therapeutic response for IMPC [16].

**Table 3:** Characteristics on breast IMPC and IDC in previous seven reports and the present study

Author	Published time	No. of cases		Component of IMPC	Criteria of eligible patients	ER and PR	Nodal metastases	Lymphovascular invasion
		IMPC	IDC					
Chen [4]	2008	100	100	Mixed	Randomly selected	Lower	Lower	\
Yu [8]	2010	72	144	Pure or more than 70 %	Age, pathologic tumor and node stage, treatment methods	Higher	Higher	Higher
Vingiani [14]	2013	49	98	Pure	Age, tumor size and grade	Higher	Higher	Higher
Liu [9]	2014	51	102	Pure	Nodal status and age	Higher	Higher	Higher
Shi [7]	2014	188	1289	mixed	Simple random sampling	Higher	Higher	Higher
Chen [10]	2014	636	297735	unknown	The same study period	Higher	Higher	\
Yu [16]	2015	267	267	Mixed	Age, pathologic tumor and node stage, treatment method	Similar	Similar	Higher
Present study	\	33	347	Mixed	The same study period	Higher	Higher	Higher

*NO* number, *IMPC* invasive micropapillary carcinoma, *IDC* invasive ductal carcinoma, *ER* estrogen receptor, *PR* progesterone receptor

There were no prospective study and only seven studies for comparative analysis between IMPC and IDC in nearly 20 years, but the criteria of eligible patients were diverse (Table 3). For example, the criteria of eligible patients in Chen’s report was “randomly selected,” the criteria of eligible patients in Yu’s report was “age, pathologic tumor and node stage, treatment method,” and so on. The criteria of eligible patients in Chen’s report was “the same study period” which was the same as our study.

It is widely agreed IMPC has its unique features such as high proclivity to lymphovascular invasion (LVI) and axillary lymph node (ALN) metastasis [7–10, 14]. However, it is controversial for survival rate of IMPC compared with IDC (Table 4).

**Table 4:** Survival on breast IMPC and IDC in previous seven reports and the present study

Author	Median follow-up (month)	OS (IMPC vs IDC)	FFS (IMPC vs IDC)	LRFS (IMPC vs IDC)	DMFS (IMPC vs IDC)
Chen [4]	60.1	59 vs 77 % *P* = 0.004	/	88.8 vs 96 % *P* = 0.055	61.2 vs 72 % *P* = 0.108
Yu [8]	45.0	86 vs 87.7 % *P* = 0.18	/	68.2 vs 81.4 % *P* = 0.045	78.1 vs 79.3 % *P* = 0.86
Vingiani [14]	51.0	89.8 vs 90.8 % *P* = 0.8	75.5 vs 79.6 % *P* = 0.47	/	/
Liu [9]	68.4	/	84.3 vs 78.4 % *P* = 0.518	/	/
Shi [7]	40.5	75.9 vs 89.5 % *P* = 0.001	67.1 vs 84.5 % *P* < 0.001	/	/
Chen [10]	48.0	82.9 vs 80.5 % *P* = 0.52	/	/	/
Yu [16]	59.0	97.7 vs 95.7 % *P* = 0.67	/	91.8 vs 96.3 % *P* = 0.03	/
Present study	39.0	97 vs 94.2 % *P* = 0.78	87.9 vs 86.2 % *P* = 0.91	93.9 vs 89.0 % *P* = 0.82	90.9 vs 89 % *P* = 0.97

*IMPC* invasive micropapillary carcinoma, *IDC* invasive ductal carcinoma, *OS* overall survival, *LRFS* local relapse-free survival, *DMFS* distant metastasis-free survival, *FFS* failure-free survival

In 2008, Chen [4] reported that the survival at 5 and 10 years in the IMPC group was significantly lower than the survival rates in the IDC group. In 2010, Yu [8] showed that the locoregional recurrence-free survival at 5 years in IMPC patients was significantly lower than that in IDC patients, but the 5-year OS and DMFS was no different between two groups. Vingiani [14] reported that disease-free survival (DFS) and OS from breast cancer for MPC and IDC patients were not statistically different in 2013. However, Vingiani’s report did not compare with Chen’s report. Vingiani’s report also did not analyze the detail between their study and Yu’s study.

In 2014, three larger retrospective studies have been reported. Liu et al. [9] showed that no difference in DFS was observed between IMPC and LN-matched IDC patients, but IMPC patients demonstrated significantly reduced survival compared to IDC patients in the T1N2–3 subpopulation, whereas IDC patients demonstrated significantly increased recurrence and metastasis compared to IMPC patients in the T2N2–3 subgroup. Chen et al. [10] showed that despite IMPC’s higher propensity for lymph node metastasis, IMPC has disease-specific survival (DSS) and overall survival (OS) that compare favorably with IDC (the 5-year rates comparing DSS and OS for IMPC was 91.8 and 82.9 %, respectively, compared with 88.6 and 80.5 % for IDC, respectively). However, Shi’s [7] results were different from Liu’s and Chen’s. Shi’s report revealed that patients with IMPC had poorer 5-year BCSS and RFS rates (75.9 and 67.1 %, respectively) than patients with IDC (89.5 %, *P* = 0.001 and 84.5 %, *P* < 0.001, respectively).

Recently, a retrospective multicenter study by Yu et al. [17] showed that the rate of distant metastasis (*P* = 0.52) and overall survival (*P* = 0.67) did not differ between the two groups. However, LRR-free survival (*P* = 0.03) and recurrence-free survival (*P* = 0.007) were significantly different between the two groups. These results were in line with their previous results in 2010 [8].

In brief, six of seven studies referred to the OS, and four of the six studies suggested that the OS of IMPC is not inferior to that of IDC. Just scattered studies provided information about RFS and DMS in the IMPC and matched series of IDC patients. Only Yu’s and Chen’s studies mentioned RFS and DMS; they revealed that IMPC showed a tendency for a higher recurrence rate and had a risk of distant metastasis similar to that observed in the matched series of IDC patients. In our study, IMPC has FFS and OS that compare similarly with IDC which is consistent with Chen’s study. However, Chen’s study did not provided information about LRFS and DMFS. We also analyzed the failure pattern and found that IMPC has LRFS and DMFS that compare similarly with IDC.

Nodal status, tumor size, tumor characteristics, and choice of surgery will dictate additional adjuvant therapies like chemotherapy, radiation, and hormonal therapy [18]. Patients with IMPC who had high percentages of ER and PR positivity, larger tumor size, greater proportion of nodal involvement, and an increased incidence of lymphovascular invasion showed no difference in survival. We thought maybe it is largely attributable to getting much more endocrine therapy, adjuvant chemotherapy, and radiotherapy than those with IDC. There are some limitations in this study. First, the number of IMPC patients was small. Second, the retrospective nature of the data introduces bias. Third, Ki-67 pathological data were not routinely obtained from patients, while Ki-67 was commonly used as a prognostic factor. In addition, longer follow-up is needed to verify the prognosis of IMPC in our study.

We believe that there will be more large-scale retrospective studies or clinical trials about IMPC of the breast to understand the prognosis. Why did the IMPC show no difference in prognosis compared with IDC though it had inferior clinical characteristics? Is it largely attributable to getting much more endocrine therapy, adjuvant chemotherapy, and radiotherapy than those with IDC? Or did IMPC have unique features of the molecular mechanisms that underlie its pathology and progression? These issues deserve our further study.

## Conclusions

Patients with IMPC had high percentages of ER and PR positivity, larger tumor size, greater proportion of nodal involvement, and an increased incidence of lymphovascular invasion. IMPC showed no difference in OS, FFS, LRFS, and DMFS compare with IDC.

## Abbreviations

DMFS, distant metastasis-free survival; ER, estrogen receptor; FFS, failure-free survival; IDC, invasive ductal carcinoma; IMPC, invasive micropapillary carcinoma; LRFS, local relapse-free survival; OS, overall survival; PR, progesterone receptor
